# Supplementary material for: Asynchronous fate decisions by single cells collectively ensure consistent lineage composition in the mouse blastocyst
Source: Nat Commun. 2016 Nov 18;7:13463. doi: 10.1038/ncomms13463 (PMC5120222; doi:10.1038/ncomms13463)
Supplement: Supplementary Information — Supplementary Figures 1-7, Supplementary Table 1. [file ncomms13463-s1.pdf]

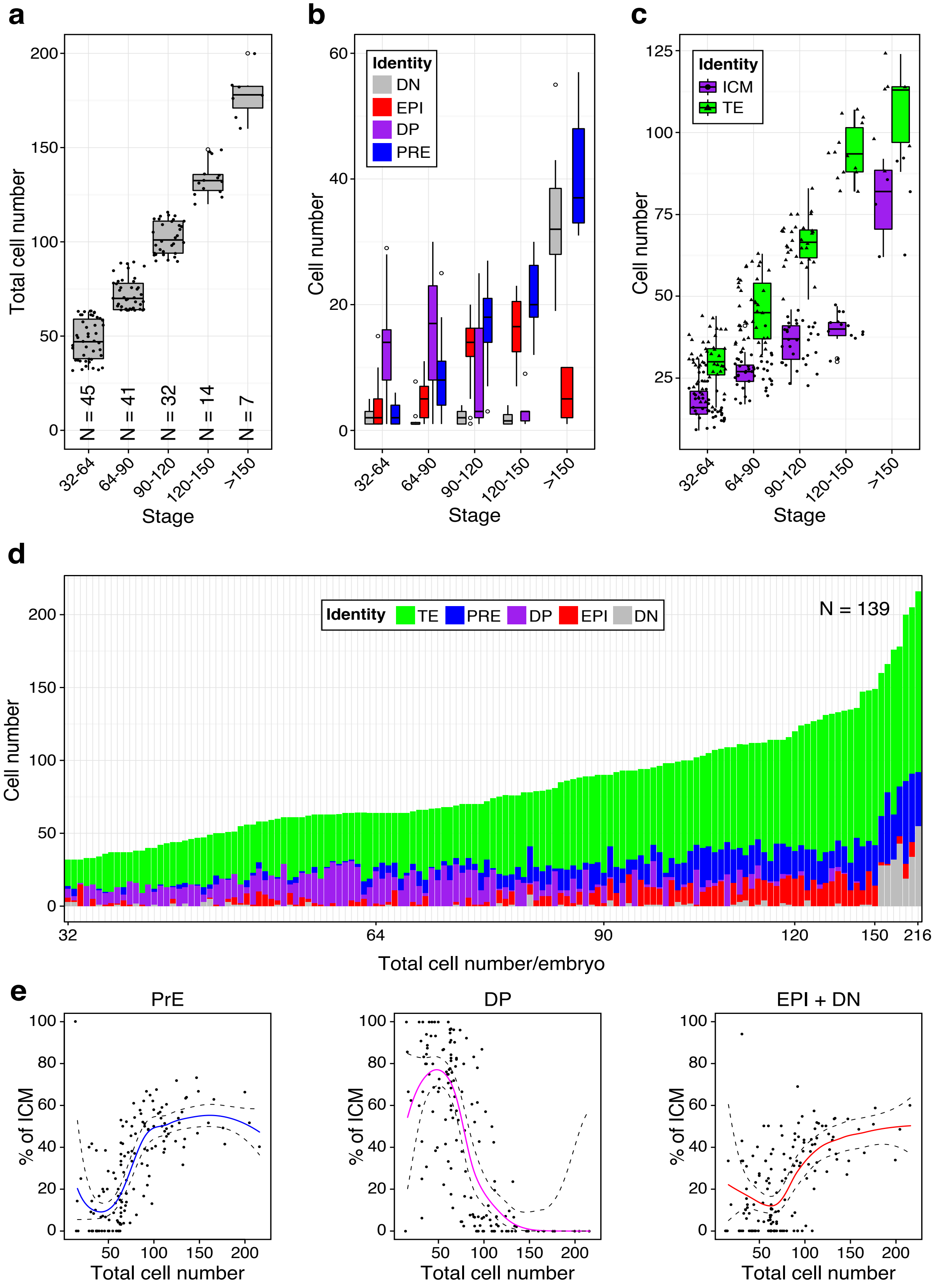

a
b
c
d
e
Total cell number
Stage
N = 45
N = 41
N = 32
N = 14
N = 7
32-64
64-90
90-120
120-150
>150
Cell number
Identity
DN
EPI
DP
PRE
ICM
TE
N = 139
Total cell number/embryo
PrE
DP
EPI + DN
% of ICM
Total cell number

Supplementary Figure 1.

Total cell counts, lineage size and lineage composition of fixed littermates (Fig. 1).

(a) Boxplot of total cell number for embryos shown in Figure 1, binned by developmental stage. Each dot represents one embryo. **(b)** Boxplot of number of cells for each ICM denomination, binned by developmental stage. Individual dots have been omitted for clarity. **(c)** Boxplot of number of TE and ICM cells for embryos shown in Figure 1, binned by developmental stage. Each dot represents the number of ICM or TE cells for one embryo. Open circles represent values outside 1.5x the inter-quartile range (IQR). **(d)** Lineage composition per embryo, shown as total number of cells, for each embryo analyzed in Fig. 1, ordered by increasing total cell number. TE and ICM identity was assigned manually. **(e)** Local regression curves showing the temporal progression in the size of each ICM lineage, as a function of the total cell number of the embryo, represented as % of the ICM. Epiblast and DN cells have been grouped together as a single lineage. Dashed lines represent confidence intervals. Each point is the % of ICM cells for that lineage in one embryo. Color-coding is indicated. TE: trophectoderm, ICM: inner cell mass, PRE: primitive endoderm (GATA6+), DP: double positive (GATA6+, NANOG+), EPI: epiblast (NANOG+), DN: double negative (GATA6-, NANOG-). For a description of the criteria used to correct fluorescence levels along the Z-axis and to determine cell identity, see Methods.

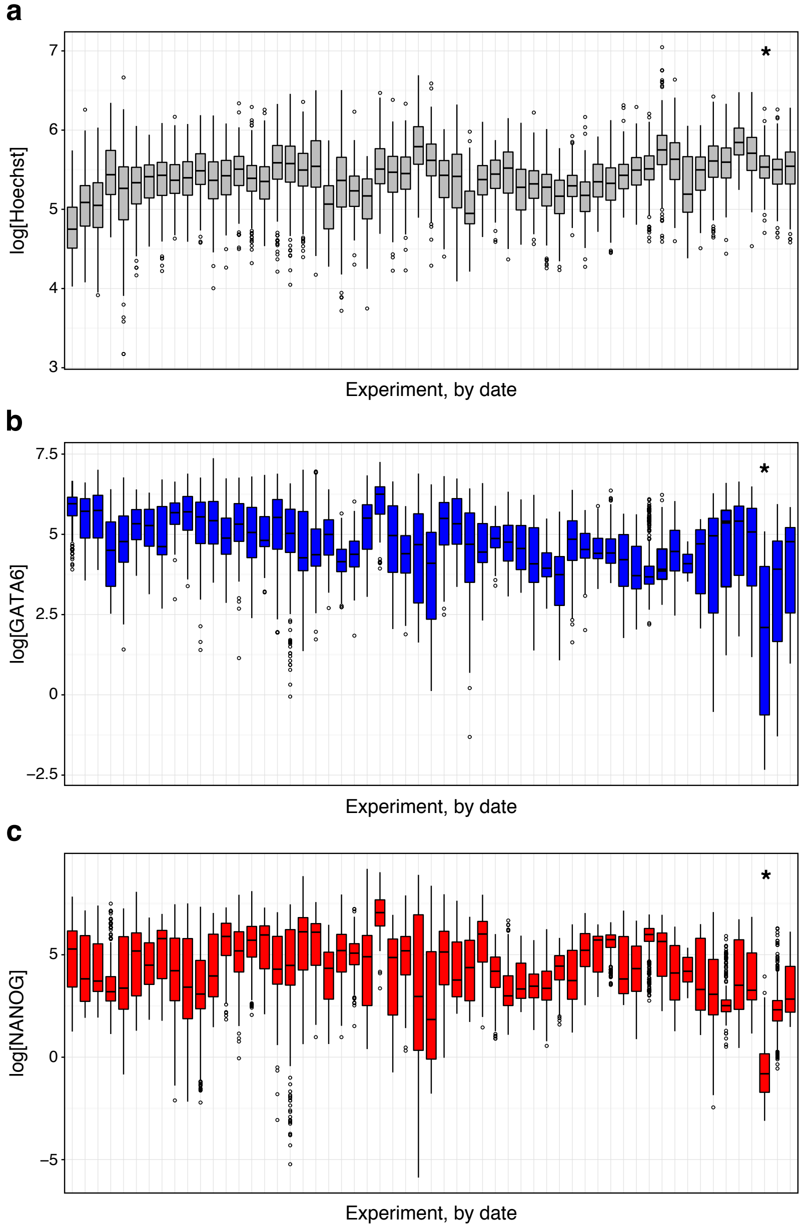

Supplementary Figure 2.
Hoechst, GATA6 and NANOG fluorescence levels per experiment.

Boxplots indicating the corrected values of fluorescence intensity (as logarithm) for each experiment in Fig. 1, 2 & 5, arranged by experimental date, in chronological order. Open circles represent values outside 1.5x the inter-quartile range (IQR). **(a)** Hoechst, **(b)** GATA6 and **(c)** NANOG. Each box and whiskers represents all ICM cells in one experiment (i.e., one single litter of embryos). The embryos in the experiment marked with an asterisk (*) were excluded from all downstream analysis because of their extremely low level of NANOG and GATA6 expression compared to equivalent embryos in other litters (2 embryos, E4.5 stage).

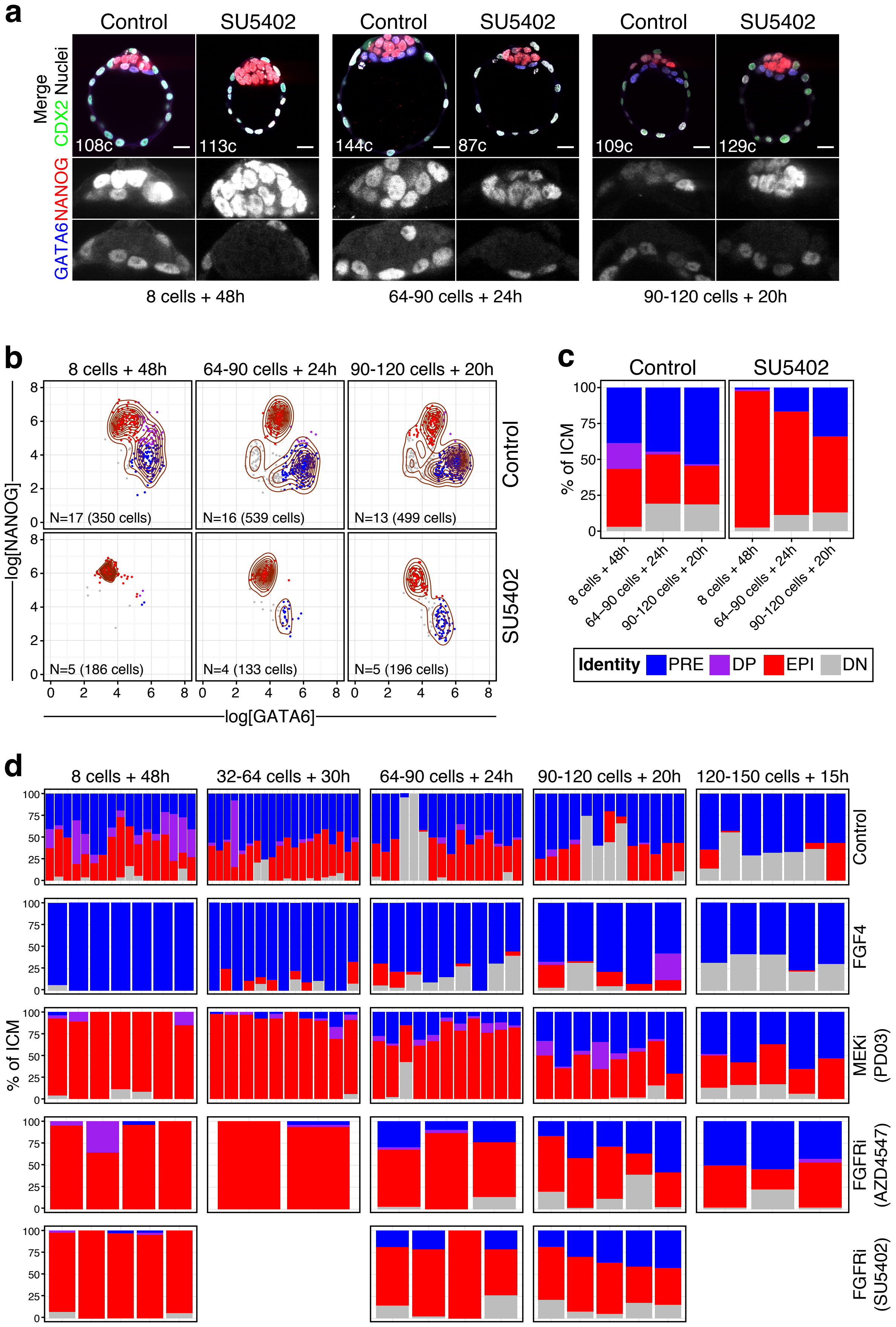
a
Control
SU5402
Control
SU5402
Control
SU5402
Merge
CDX2 Nuclei
NANOG
GATA6
108c
113c
144c
87c
109c
129c
8 cells + 48h
64-90 cells + 24h
90-120 cells + 20h
b
8 cells + 48h
64-90 cells + 24h
90-120 cells + 20h
log[NANOG]
log[GATA6]
N=17 (350 cells)
N=16 (539 cells)
N=13 (499 cells)
N=5 (186 cells)
N=4 (133 cells)
N=5 (196 cells)
Control
SU5402
c
Control
SU5402
% of ICM
8 cells + 48h
64–90 cells + 24h
90-120 cells + 20h
Identity
PRE
DP
EPI
DN
d
8 cells + 48h
32-64 cells + 30h
64-90 cells + 24h
90-120 cells + 20h
120-150 cells + 15h
Control
FGF4
% of ICM
MEKi (PD03)
FGFRi (AZD4547)
FGFRi (SU5402)

Supplementary Figure 3.

Embryos treated with SU5402 and ICM composition per embryo for treated embryos in Fig. 2. **(a)** Representative immunofluorescence images of embryos treated in the conditions and regimes indicated. Control: KSOM; SU5402: KSOM + 20μM SU5402. Treatment regimes are like those indicated in Fig. 2a. NANOG (epiblast) and GATA6 (PrE) are shown in grayscale in ICM magnifications. In merged image, CDX2 marks the TE lineage and "c" indicates the total number of cells of the embryo shown. All images are 5 μm Z-projections. **(b)** Scatter plots for the levels of GATA6 and NANOG (expressed as logarithm) in individual ICM cells of embryos treated as in (a). Columns represent treatment regimes and rows represent treatment conditions. Contour lines have been overlaid as density estimators. Cell identity (color-coded) was assigned using the same clusters used for embryos in Fig. 2b. Number of embryos (N) and cells analyzed are indicated in each plot. **(c)** Average ICM composition at the end of the culture period for embryos treated in each of the conditions and regime (indicated on the X-axis), shown as % of the ICM. **(d)** ICM composition, per embryo, for all embryos analyzed in (b-c) and Fig. 2, shown as % of the ICM. Columns represent treatment regimes, as described in Fig. 2a and rows represent treatment conditions. Color-coding is indicated. PRE: primitive endoderm (GATA6+), DP: double positive (GATA6+, NANOG+), EPI: epiblast (NANOG+), DN: double negative (GATA6-, NANOG-); FGF4: fibroblast growth factor-4, MEKi: MAPK/ERK kinase (MEK) inhibitor, FGFRi: FGF receptor inhibitor. For a description of the criteria used to correct fluorescence levels along the Z-axis and to determine cell identity, see Methods. Scale = 20 μm.

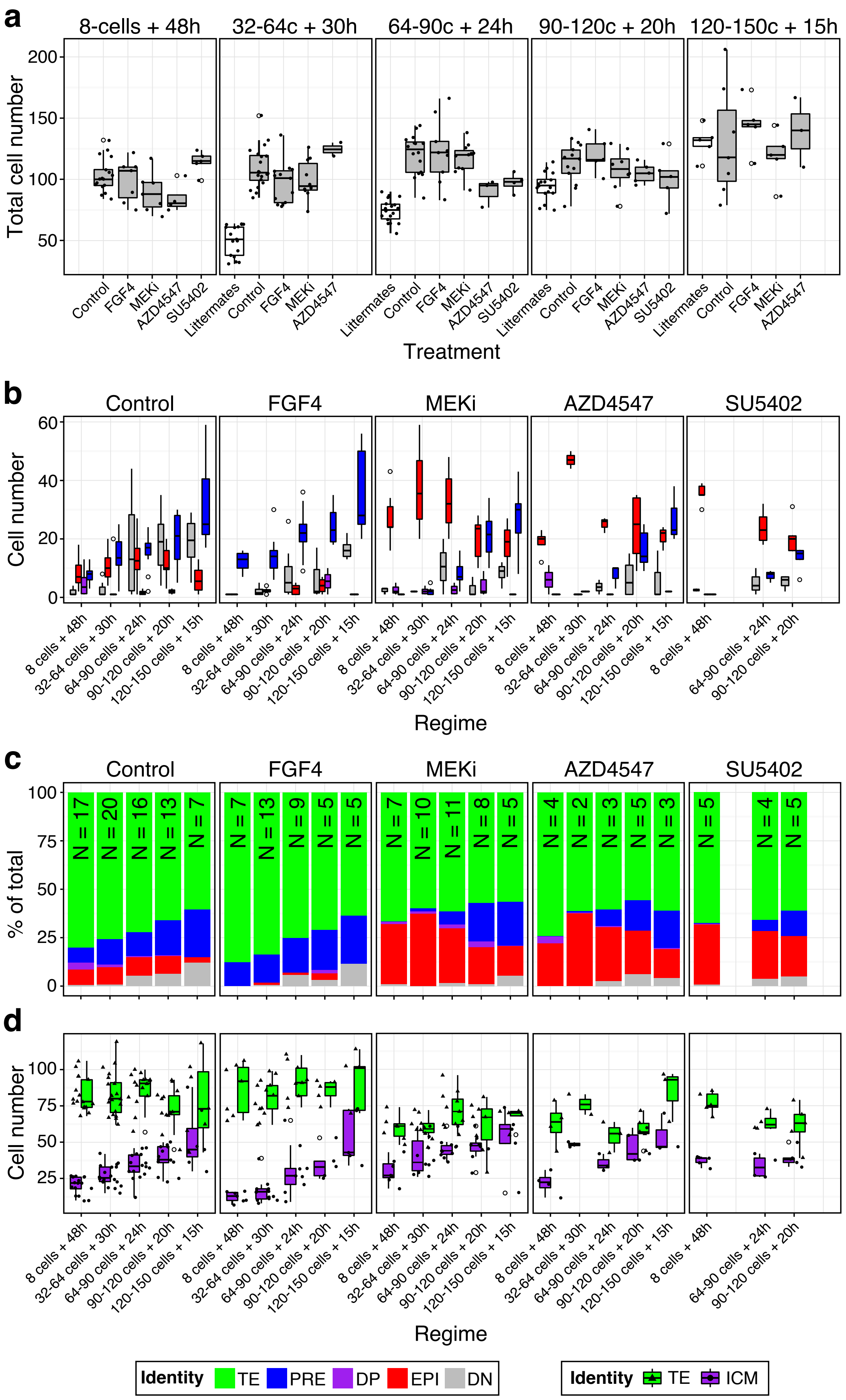

a
8-cells + 48h
32-64c + 30h
64-90c + 24h
90-120c + 20h
120-150c + 15h
Total cell number
Treatment
Control
FGF4
MEKi
AZD4547
SU5402
Littermates
b
Control
FGF4
MEKi
AZD4547
SU5402
Cell number
Regime
8 cells + 48h
32-64 cells + 30h
64-90 cells + 24h
90-120 cells + 20h
120-150 cells + 15h
c
% of total
N = 17
N = 20
N = 16
N = 13
N = 7
N = 7
N = 13
N = 9
N = 5
N = 5
N = 7
N = 10
N = 11
N = 8
N = 5
N = 4
N = 2
N = 3
N = 5
N = 3
N = 5
N = 4
N = 5
d
Cell number
Regime
Identity TE PRE DP EPI DN
Identity TE ICM

Supplementary Figure 4.

Total cell counts, lineage size and lineage composition of treated embryos in Fig. 2.

(a) Boxplots of total cell number for embryos shown in figures 2, 3 and Supplementary Fig. 3, binned by treatment condition and grouped by treatment regime. White boxes correspond to reference littermates for each group (fixed at the time of collection – these embryos are also shown in Figure Supplementary Fig. 1a). Gray boxes indicate the cell number of treated embryos, at the end of the culture period, for each treatment condition. Control: KSOM or KSOM + 1 μg/ml of heparin (addition of heparin had no detectable effect on control embryos); FGF4: KSOM + 1 μg/ml rhFGF4 + 1 μg/ml of heparin; MEKi: KSOM + 1 μM PD0325901; AZD4547: KSOM + 1 μM AZD4547; SU5402: KSOM + 20 μM SU5402. **(b)** Boxplots of number of cells for each ICM lineage for embryos shown in figures 2, 3 and Supplementary Fig. 3, binned by treatment regime and grouped by treatment condition. Individual dots have been omitted for clarity. **(c)** Average lineage composition, shown as % of the total, for embryos shown in figures 2, 3 and Supplementary Fig. 3, binned by treatment regime and grouped by treatment condition. **(d)** Boxplots of number of TE and ICM cells for embryos shown in (c). Each dot represents the number of TE or ICM cells for one embryo. Open circles represent values outside 1.5x the inter-quartile range (IQR). Color-coding is indicated. TE: trophectoderm, ICM: inner cell mass, PRE: primitive endoderm (GATA6+), DP: double positive (GATA6+, NANOG+), EPI: epiblast (NANOG+), DN: double negative (GATA6-, NANOG-), FGF4: fibroblast growth factor-4, MEKi: MAPK/ERK kinase (MEK) inhibitor. For a description of the criteria used to correct fluorescence levels along the Z-axis and to determine cell identity, see Methods.

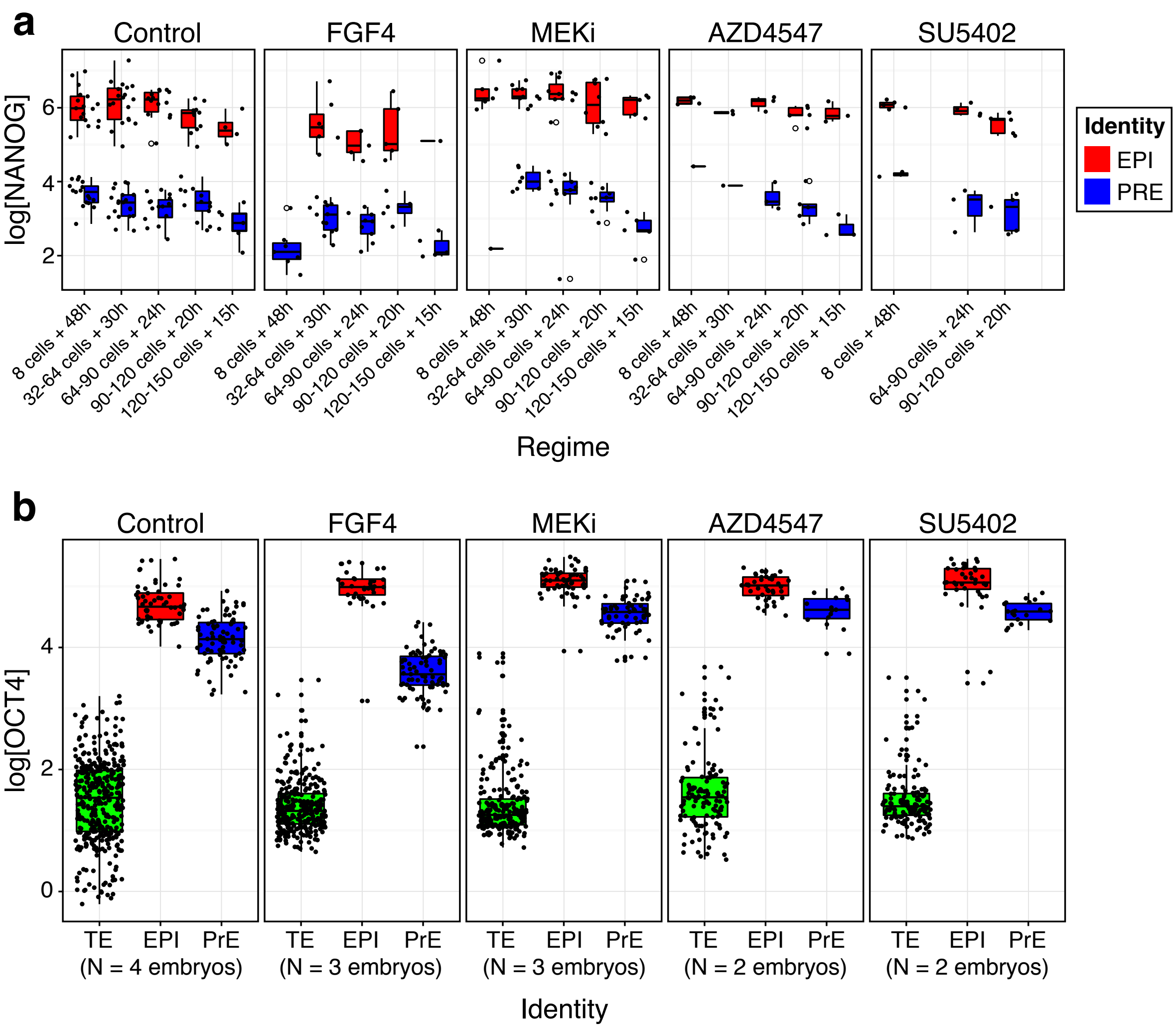

Supplementary Figure 5.

NANOG and OCT4 levels for embryos in Figs. 2 and 3.

(a) Boxplots of NANOG levels (as logarithm) for embryos shown in Fig. 2, binned by treatment regime and grouped by treatment condition. Each dot represents the average log[NANOG] for one embryo, for all cells in the corresponding lineage. Open circles represent values outside 1.5x the inter-quartile range (IQR). Color-coding is indicated. N numbers are given in Supplementary Fig. 4. **(b)** Boxplots of OCT4 levels (as logarithm) in each lineage of embryos shown in figure 3, grouped by treatment condition. Each dot represents a single epiblast, PrE or TE cell, as indicated. TE cells are shown for reference, as they show no, or negligible, OCT4 expression. TE: trophectoderm, ICM: inner cell mass, PRE/PrE: primitive endoderm, EPI: epiblast, FGF4: fibroblast growth factor-4, MEKi: MAPK/ERK kinase (MEK) inhibitor.

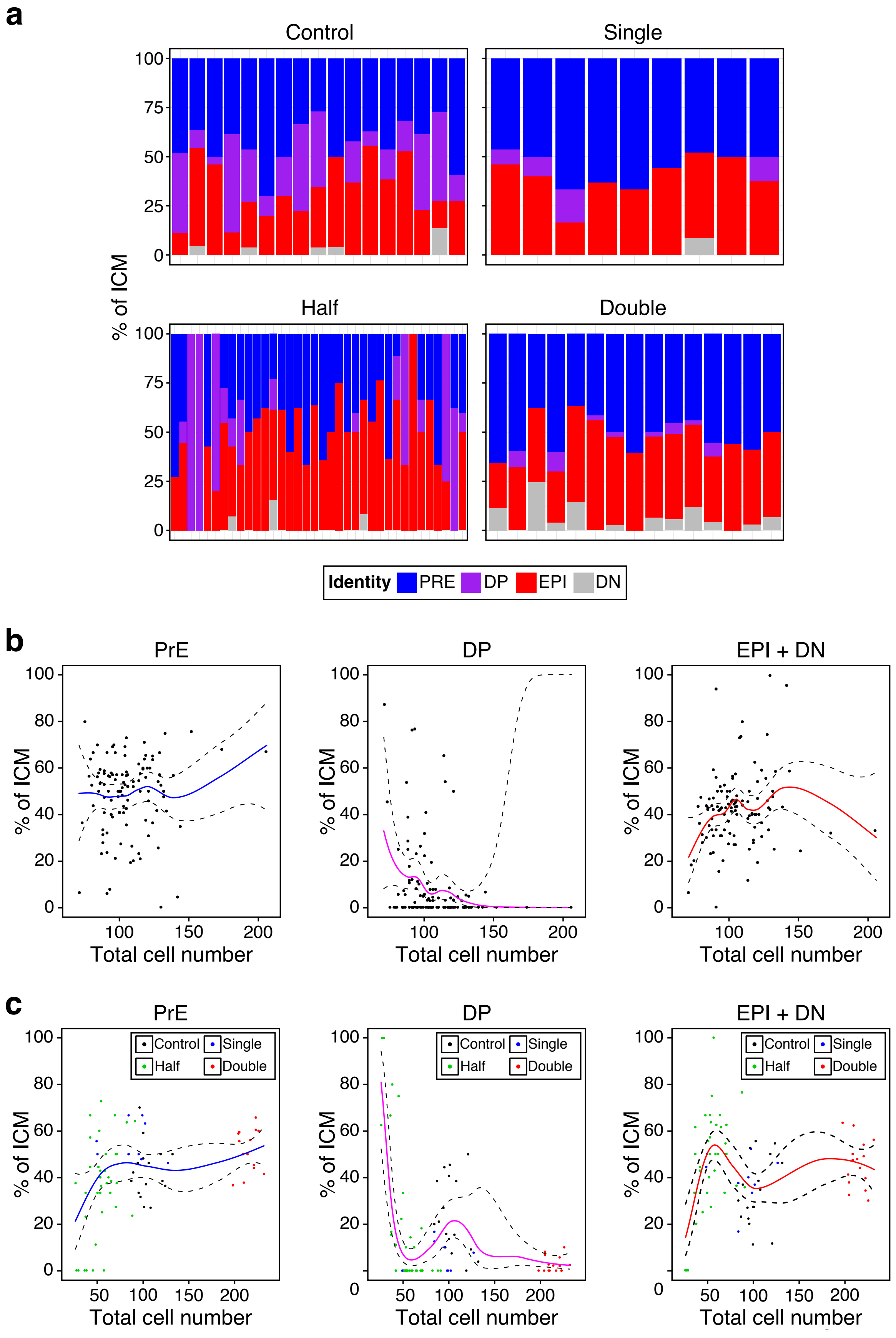

a
Control
Single
Half
Double
% of ICM
Identity PRE DP EPI DN
b
PrE
DP
EPI + DN
% of ICM
Total cell number
c
PrE
DP
EPI + DN
Control Single Half Double
% of ICM
Total cell number

Supplementary Figure 6.

ICM composition per embryo for embryos in Figs. 4 and 2.

(a) ICM composition per embryo at the end of the culture period for control, single, half and double embryos shown in Fig. 4i, as % of the ICM. Each bar represents a single embryo. **(b)** Local regression curves showing the size of each ICM lineage in control embryos (for all regimes shown in Fig. 2a), as a function of the total cell number of the embryo (most embryos have between 100-170 cells), represented as % of the ICM. **(c)** Local regression curves showing the size of each ICM lineage in control (black), single (blue), half (green) and double (red) embryos (shown in Figs. 4 and in (a)), as a function of the total cell number of the embryo, represented as % of the ICM. Epiblast and DN cells have been grouped together as a single lineage. Dashed lines represent confidence intervals. Each point is the % of ICM cells for that lineage in one embryo. Color-coding is indicated. PRE: primitive endoderm (GATA6+), DP: double positive (GATA6+, NANOG+), EPI: epiblast (NANOG+), DN: double negative (GATA6-, NANOG-).

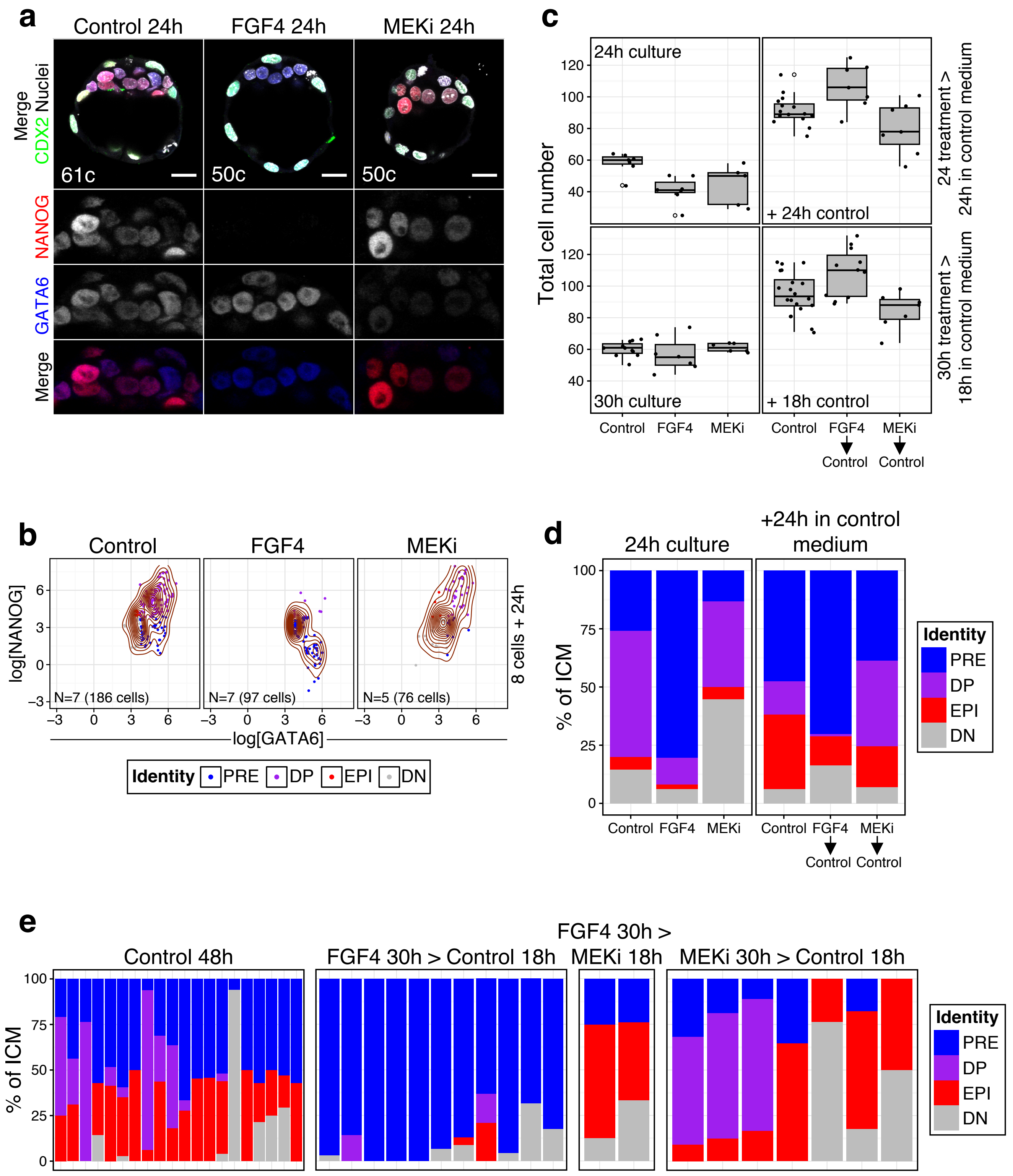
a
Control 24h
FGF4 24h
MEKi 24h
Merge
CDX2 Nuclei
61c
50c
50c
NANOG
GATA6
Merge
b
Control
FGF4
MEKi
log[NANOG]
log[GATA6]
N=7 (186 cells)
N=7 (97 cells)
N=5 (76 cells)
8 cells + 24h
Identity PRE DP EPI DN
c
24h culture
+ 24h control
30h culture
+ 18h control
Total cell number
24 treatment > 24h in control medium
30h treatment > 18h in control medium
Control FGF4 MEKi
Control FGF4 MEKi
Control Control
d
24h culture
+24h in control medium
% of ICM
Identity
PRE
DP
EPI
DN
e
Control 48h
FGF4 30h > Control 18h
FGF4 30h > MEKi 18h
MEKi 30h > Control 18h
% of ICM

Supplementary Figure 7.

24h FGF4-RTK-MAPK modulation, total cell numbers and ICM composition per embryo for embryos in Fig. 5.

(a) Representative immunofluorescence images of embryos cultured in the conditions indicated for 24h. NANOG (epiblast) and GATA6 (PrE) are shown in grayscale in ICM magnifications and color-coded in the merged panels. In top merged image, CDX2 marks the TE lineage and "c" indicates the total number of cells of the embryo shown. All images are 5 μm Z-projections. **(b)** Scatter plots for the levels of GATA6 and NANOG (expressed as logarithm) in individual ICM cells, for each treatment condition after 24 in culture, as in (a). Contour lines have been overlaid as density estimators. Cell identity (color-coded) was assigned using the same clusters used for embryos in Fig. 1d and 5b. Number of embryos (N) and cells analyzed are indicated in each panel. **(c)** Boxplots of total cell number of embryos shown here and in figure 5, binned by treatment condition and grouped by experimental point (columns) and treatment regime (rows). **(d)** Average ICM composition for embryos cultured in each of the conditions indicated for 24h or cultured for 24h and then transferred to control medium for a further 24h, as indicated. **(e)** ICM composition (as % of ICM) for all embryos shown in Fig. 5f at the end of the culture conditions indicated. Each bar represents a single embryo. Control: KSOM or KSOM + 1 μg/ml of heparin (addition of heparin had no detectable effect on control embryos); FGF4: KSOM + 1 μg/ml rhFGF4 + 1 μg/ml of heparin; MEKi: KSOM + 1 μM PD0325901. Color-coding is indicated.. DN: double negative (GATA6-, NANOG-), DP: double positive (GATA6+, NANOG+), PRE: primitive endoderm (GATA6+), DP: double positive (GATA6+, NANOG+), EPI: epiblast (NANOG+), DN: double negative (GATA6-, NANOG-), FGF4: fibroblast growth factor-4, MEKi: MAPK/ERK kinase (MEK) inhibitor. For a description of the criteria used to correct fluorescence levels along the Z-axis and to determine cell identity, see Methods. Scale = 20 μm.

Supplementary Table 1.
Summary of treatment regimes used.

Stage of collection	Treatment length*	Stage at fixation	Regime shorthand
8-cell	48h	120-170 cells	Regime 1 (R1)
8-cell	24h treatment + 24h control	120-170 cells	Regime 8 (R8)
8-cell	30h treatment + 18h control	120-170 cells	Regime 9 (R9)
32-64 cells	30h	120-170 cells	Regime 5 (R5)
64-90 cells	24h	120-170 cells	Regime 3 (R3)
90-120 cells	20h	120-170 cells	Regime 4 (R4)
120-150 cells	15h	120-170 cells	Regime 6 (R6)

* Exact treatment length may have varied (longer, or shorter), depending on the stage of collection, as regimes were only definitely assigned retrospectively.
